# Duplicate gene evolution, homoeologous recombination, and transcriptome characterization in allopolyploid cotton

**DOI:** 10.1186/1471-2164-13-302

**Published:** 2012-07-06

**Authors:** Lex E Flagel, Jonathan F Wendel, Joshua A Udall

**Affiliations:** 1 Department of Biology, Duke University, Box 90338, Durham, NC 27708, USA; 2 Department of Ecology, Evolution, and Organismal Biology, Iowa State University, Ames, IA 50014, USA; 3 Department of Plant and Wildlife Sciences, Brigham Young University, Provo, Utah 84602, USA

**Keywords:** Cotton, Duplicate gene evolution, Gene conversion, *Gossypium*, Homoeologous recombination, Polyploidy, Transcriptome

## Abstract

**Background:**

Modern allotetraploid cotton contains an “A” and “D” genome from an ancestral polyploidy event that occurred approximately 1–2 million years ago. Diploid A- and D-genome species can be compared to the A- and D-genomes found within these allotetraploids to make evolutionary inferences about polyploidy. In this paper we present a comprehensive EST assembly derived from diploid and model allotetraploid cottons and demonstrate several evolutionary inferences regarding genic evolution that can be drawn from these data.

**Results:**

We generated a set of cotton expressed sequence tags (ESTs), comprising approximately 4.4 million Sanger and next-generation (454) transcripts supplemented by approximately 152 million Illumina reads from diploid and allotetraploid cottons. From the EST alignments we inferred 259,192 genome-specific single nucleotide polymorphisms (SNPs). Molecular evolutionary analyses of protein-coding regions demonstrate that the rate of nucleotide substitution has increased among both allotetraploid genomes relative to the diploids, and that the ratio of nonsynonymous to synonymous substitutions has increased in one of the two polyploid lineages we sampled. We also use these SNPs to show that a surprisingly high percentage of duplicate genes (~7 %) show a signature of non-independent evolution in the allotetraploid nucleus, having experienced one or more episodes of nonreciprocal homoeologous recombination (NRHR).

**Conclusions:**

In this study we characterize the functional and mutational properties of the cotton transcriptome, produce a large genome-specific SNP database, and detect illegitimate genetic exchanges between duplicate genomes sharing a common allotetraploid nucleus. Our findings have important implications for our understanding of the consequences of polyploidy and duplicate gene evolution. We demonstrate that cotton genes have experienced an increased rate of molecular evolution following duplication by polyploidy, and that polyploidy has enabled considerable levels of nonreciprocal exchange between homoeologous genes.

## Background

Cotton produces the world’s most utilized natural fiber, making it a staple of the textile and garment industries. In 2009 cotton fiber production in the US was valued at approximately 3.74 billion dollars, with an additional 666 million dollars derived from cotton seed extracts [1]. This places cotton among the top five most valuable US crops. Accordingly, it is important to understand cotton’s gene repertoire and its evolutionary history. With the vast majority of cotton production being derived from the allotetraploid species *Gossypium hirsutum*, followed by smaller contributions from a second domesticated allotetraploid, *G. barbadense*, an appreciation of cotton biology in the context of its allotetraploid history has become a major research focus for the improvement of this crop. Here we present a comprehensive assembly of approximately 4.4 million EST sequences from these two allotetraploid species and from the diploid species representing the progenitor genomes of allotetraploid cotton. We illustrate how this assembly facilitates diagnosis of the genome-of-origin for what otherwise would have been ambiguously derived transcripts, and demonstrate the utility of this assembly for addressing questions pertaining to the phenomenon of allopolyploidy and cotton transcriptome evolution.

The cotton genus comprises about 50 species, divided into eight monophyletic diploid genome groups (historically denoted alphabetically as A through G, and K), and a clade of five allotetraploid species, the latter containing a genome from both the A and D diploid genome groups (with allotetraploids denoted as the AD group). The cytological and phylogenetic history of this genus is well-resolved (reviewed in: [2-5]), and data demonstrate that the best living models of the progenitor diploids of the A- and D-genomes of modern allotetraploid cotton are the diploid species *G. arboreum* and *G. raimondii*, respectively. In addition, extensive seed collections are available and include both wild progenitor forms along with their domesticated descendants. Collectively, these resources provide a useful system for studying the genomic and phenotypic changes that accompany domestication. Embedding these resources within their phylogenetic context results in a powerful framework for the study of polyploidy and domestication, and consequently cotton has emerged as a model system in these areas of research. Recent examples of the utility of the cotton system in this regard include detailed analyses of the cotton fiber transcriptome during development [6-10], as well as explorations of the mutational and expression changes that accompany allopolyploidization [11-19]. These types of studies, as well as those aimed at crop improvement, are enriched or facilitated by the availability of cotton EST collections, underscoring the utility and importance of developing a rich and deep database of EST resources.

Prior to this study, the community-driven global assembly of cotton ESTs included ~ 170,000 Sanger sequences, with ~ 31,000 *G. arboreum* (A-diploid model progenitor), ~ 69,000 *G. raimondii* (D-diploid model progenitor), and ~ 70,000 *G. hirsutum* (AD allotetraploid) sequences assembled into a single reference collection yielding 51,107 contigs [20]. Because this assembly (and subsequent incremental updates) contained sequences from both model diploid parents and a natural allotetraploid representing their combination, it could be used to identify A- and D-genome-specific SNPs, which can in turn be used to assign genomic origin to homoeologous transcripts in allotetraploid cotton (*homoeologs* are duplicate genes originating via polyploidy). Implementation of this EST collection strategy has enabled several research possibilities, including monitoring genome-specific expression in natural allotetraploids [14,16,19,21-23] and the quantification of the extent of nonreciprocal homoeologous recombination among natural allotetraploid species (i.e. recombination between homoeologs of the A- and D-genomes in an AD allotetraploid) [18]. These efforts have elucidated the dynamics of gene expression evolution and genic content variation that accompanied allotetraploid formation and evolution in cotton.

Here we present a vastly expanded cotton EST assembly, which contains approximately 4.4 million Sanger and next-generation (454) transcripts. Like previous assemblies [20], this one incorporates ESTs from both the A- and D-genome diploid progenitors, along with allotetraploid ESTs from two species of allotetraploid cotton, *G. barbadense* and *G. hirsutum*. The 56,373 contigs obtained from this assembly represent a vastly expanded representation of the genic content of cotton. To add additional depth to the assembly, we also generated ~152 million 82 bp Illumina reads, representing the fiber transcriptome of diploid A- and D-genome cotton as well as the allotetraploids *G. barbadense* and *G. hirsutum*. Together these resources allow us to detect 259,192 genome-specific SNPs, which in turn can be used to distinguish the A- and D-genome homoeologs found in the allotetraploid cotton genome.

We describe this collection and document its utility for genome-specific transcriptome analysis in allotetraploid cotton. We also present a characterization of the functional properties of the cotton transcriptome and analyses of molecular evolution following the most recent whole genome duplication that accompanied allotetraploid formation 1–2 million years ago[3,5]. At the time of writing, allotetraploid cotton is now among the most important crops lacking a whole genome sequence, but as progress is made in this regard, the EST assembly and genome-specific SNP resources presented here will be of use in assembling and annotating the cotton genome.

## Results

### EST assembly characteristics

The cotton assembly presented here contains EST sequences (~1.35 Gigabases) from *G. arboreum* (A-genome), *G. barbadense* (AD-genome), *G. hirsutum* (AD-genome), and *G. raimondii* (D-genome) (Table 1). ESTs from these species were assembled jointly in a multispecies assembly. The justification for this multispecies assembly stems from the fact that the transcripts from resident A- and D-genomes in the allotetraploid nucleus are more closely related to their diploid homologs than they are to each other ([13] and see below). Thus, combining diploid EST sequences with the ESTs of two tetraploid species does not introduce appreciable amounts of additional sequence divergence. From a total of approximately 4.9 million *Gossypium* ESTs, 4,395,458 were assembled into contigs, while 510,570 ESTs remained unassembled (i.e. singletons). The assembly includes previously published Sanger sequences ([20]; and references therein) as well as approximately 4.66 million new 454 sequences (Table 1). The assembled collection of ESTs produced 56,373 contigs with an average length of 1,016.5 bp (min = 45, max = 9,648). A study that validates the quality of this assembly is described in the *Materials and Methods* section, wherein we find high nucleotide similarity between our assembled contigs and cloned and sequenced cotton genes. This suggests that the sequence quality and assembly routine accurately recapitulate the structure and mutational history found in well-characterized cotton genes.

**Table 1:** Tissues, sequence platform, and number of reads generated for each of the four cotton species used in this study

**Species *(accession)***	**Designation**	**Tissues in library**	**Type of sequence**	**Approx. number of ESTs (X 1000)**	**UnassembledESTs (X 1000)**
*G. arboreum* (*cv.* AKA8401)	A_2_	gynoecium, calyx, fiber, roots, whole seedlings	454-FLX	692	39
*G. arboreum*^1^	A_2_	cotton fiber 7–10 dpa	Sanger	28	3
*G. raimondii* (GN33)	D_5_	gynoecium, calyx, fiber, roots, whole seedlings	454-FLX	710	71
*G. raimondii* (GN33)	D_5_	meristem, calyx, fiber, root, petal, seedling	454-Titanium	745	33
*G. raimondii* (GN33)	D_5_	whole seedling, normalized floral organs including developing embryos	Sanger	57	8
*G. hirsutum* (*cv.* Maxxa*)*	AD_1_	gynoecium, calyx, fiber, roots, whole seedlings	454-FLX	607	58
*G. hirsutum* (Tx2094*)*	AD_1_	bud, leaf, stem, whole seedling (with roots)	454-Titanium	830	92
*G. hirsutum*^1^	AD_1_	various sources. ESTs that are publically available in GenBank	Sanger	250	35
*G. barbadense* (*cv.* Pima S6)	AD_2_	bud, leaf, stem, whole seedling (with roots)	454-Titanium	861	90
*G. barbadense* (K101)	AD_2_	bud, leaf, stem, whole seedling (with roots)	454-Titanium	713	82
*G. barbadense*^1^	AD_2_	various sources. ESTs that are publically available in GenBank	Sanger	1	0

^1^Accession(s) unknown or derived from numerous accessions. References for Sanger EST collections can be found in Udall *et al.*[20].

Among the contigs, 48,729 include a predicted coding region, with an average coding length of 863.0 bp. This estimated coding sequence length for cotton is a little less than the average coding sequence length of some angiosperms with sequenced genomes. For example, the average coding sequence lengths of *A. thalianaPopulus trichocarpa* (poplar), and *Vitis vinifera* (grape) are 1209.3, 1115.9, and 1072.3 bp, respectively (retrieved from Phytozome v5.0 [24]). If we assume that the true coding sequence length in cotton is comparable to the average length for these species (1132.5 bp), we estimate that our EST assembly provides coverage of about 76% of the coding sequence for the average contig. In addition to breadth of coverage, the assembly achieves a new level of depth of coverage; the median number of ESTs per contig was 44, with a range from 2 to 2,167, and the average depth of coverage for each assembled nucleotide was 23.6. These results mark a significant improvement over previous cotton transcriptome assemblies, where the average contig length was 791 bp, the median number of ESTs per contig was 3, and the average depth of coverage for each assembled nucleotide was 3.4 [20].

**Figure 1:**
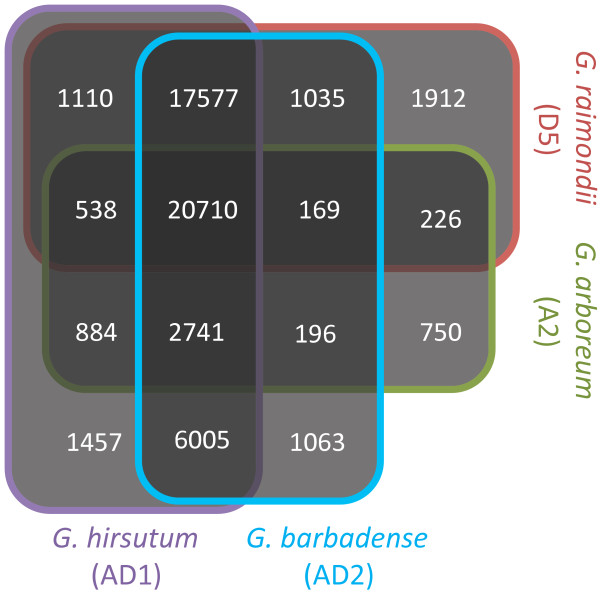
**Venn Diagram of EST content for the four cotton species included in this study.** The value in each box represents the number of contigs that include at least one EST from a particular combination of species.

To assess our expectation of co-assembly of expressed sequences from multiple cotton genomes, species membership was compiled for each contig of the assembly (Figure 1). The most common class of contigs (n = 20,710) are those that contain ESTs from all four species (*G. arboreum*, *G. barbadense*, *G. hirsutum*, and *G. raimondii*), indicating that genic conservation is substantial among these species and that we have achieved sequence coverage sufficient to reveal this. 21,643 contigs were represented by sequences from both *G. arboreum* (A-genome) and *G. raimondii* (D-genome), whereas 47,033 contigs contained sequences derived from both allotetraploids.

Interestingly, we observed 17,577 contigs for which sequences were lacking only for *G. arboreum* and only 750 contigs constructed entirely from *G. arboreum* ESTs (Figure 1). These findings together suggest that the *G. arboreum* ESTs collection represents a significantly narrower pool of genes relative to the other species. Although it is possible that some *G. arboreum* orthologs are missing because of gene loss or gene silencing, there is substantial evidence that *G. arboreum* has not lost a large portion of its transcriptome or experienced massive global shifts in gene expression [7,11,19,20,25]. We note that *G. arboreum* was the only species lacking 454 sequences generated by Titanium chemistry, having instead only 454 FLX reads. In support of this point, we do observe a 13 % enrichment of 454 Titanium reads from *G. barbadenseG. hirsutum*, and *G. raimondii* in the contigs missing only *G. arboreum* when compared to their proportions in all other contigs (data not shown). Our best hypothesis is that technical differences between these two sequencing chemistries, including sequencing bias, differences in library construction, and assembly characteristics underlie the unexpectedly narrow breadth of the *G. arboreum* EST collection.

### Assembly annotation

Sequence similarity searches (BLASTX) to the RefSeq (NCBI) database were used to provide an approximation of gene function. The contigs and singletons (>300 bp) were compared to annotated proteins from RefSeq with a minimum e-value score of 1e^-15^. 6,763 contig sequences had a high similarity (e-value = 0) to RefSeq sequences and 43,336 contig sequences had a match less than 1e^-15^. The contig sequences were also compared to the annotated genomes of *Carica papaya* and *A. thaliana*. When only the top match was considered, 4,984 contigs had a near identical match to an *A. thaliana* gene (e-value = 0) and 17,419 contigs had top matches with an e-value lower than 1e^-100^. More than 43,000 contigs had a top match to an *A. thaliana* gene with an e-value lower than 1e^-15^. When only the top *C. papaya* match was considered, 4,844 contigs had a near identical match to a *C. papaya* gene (e-value = 0) and 16,569 had top matches with an e-value lower than 1e^-100^. More than 40,000 contigs had a top match to a *C. papaya* gene with an e-value lower than 1e^-15^. The similar results obtained with both *Arabidopsis* and *Carica* lend confidence that this set of annotations is accurate.

### Identification of protein coding domains

Among the 56,373 contigs in the assembly, 48,729 had a predicted translational product (assessed using ESTScan [26]), and among this subset 31,921 protein products had at least one annotated Pfam domain or could be assigned to a Pfam family. Not surprisingly, the most abundant domain was the Pentatricopeptide repeat (PPR; Pfam: PF01535; Additional file 1). This domain is among the most numerous in the angiosperms and may be associated with RNA stabilization and processing [27]. Other highly prevalent Pfam annotations include the WD40, protein kinase, and Myb DNA binding domains, as well as the leucine rich repeat and RNA recognition motifs. Like the PPR repeat, these domains are also abundant in other angiosperm genomes.

**Figure 2:**
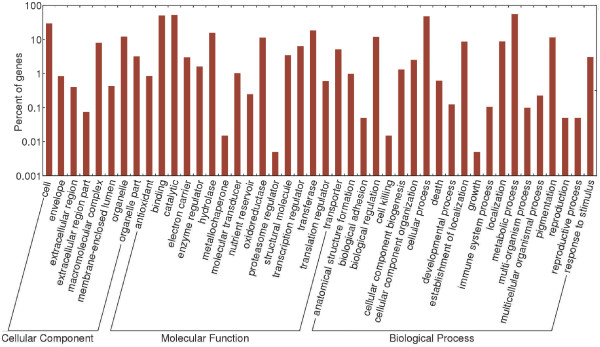
**Distribution of high-level Gene Ontology (GO) categories among all contigs.** Components of each of the three top-level GO categories (Cellular Component, Molecular Function, and Biological Process) are indicated.

To better assess the functional characteristics of the cotton transcriptome assembly, we converted Pfam annotations into GO categories. The relative abundance of various high-level GO categories is shown in Figure 2. These data indicate that our current cotton EST assembly includes a large and diverse set of genes. This broad sampling of annotated genes should be useful for enrichment-based tests among differentially expressed genes as determined by future microarray, RNA-Seq, or similar experiments. We anticipate this set of genes will serve as a useful transcriptome model until empirically derived transcripts can be paired with gene predictions from a *de novo* cotton genome sequence.

### Homoeologous SNPs

Because our cotton EST assembly includes a large number of sequences from A- and D-genome diploid progenitors as well as AD-genome allotetraploid derived ESTs, it can be used to diagnose homoeologs and homoeolog-specific SNPs. In a strategy similar to Buggs *et al.*, [28], we identified homoeolog-specific SNPs directly from the 454/Sanger assembly and supplemented this assessment with 151,863,736 Illumina sequences, also derived from the A- and D-genome diploid progenitors and AD-genome allotetraploids, and mapped to the *de novo* 454/Sanger EST assembly. This strategy leverages the strengths of both the 454 and Illumina next-generation platforms. The 454 (and legacy Sanger) reads are longer and assemble into contigs more readily, while the Illumina platform offers deep sequence coverage that can be used to confirm the 454/Sanger SNPs and find new SNPs in regions with low 454/Sanger read coverage.

In total, we identified 259,192 SNPs between the diploid A- and D-genomes, including 186,464 that were exclusive to the *de novo* 454/Sanger assembly, 122,940 exclusive to the Illumina reference mapping, and 50,212 common to both strategies (Additional file 2). The intersection of both SNP pools is only a fraction of the total. However, to identify homoeologous SNPs in the 454/Sanger assembly it is necessary to have ESTs from the A- and D-genome diploids present, as is the case for 21,643 contigs (Figure 1). Furthermore, within these contigs the A- and D-genome diploid sequences must overlap one another. This occurs for approximately 29 % of all available nucleotide positions within the eligible contigs (data not shown). Finally, cross-validating a SNP with both the 454/Sanger assembly and the Illumina reads requires an overlap between both diploid parents from both sources of ESTs (454/Sanger and Illumina ESTs). This occurs for about 23% of all sites from the 21,643 eligible contigs (data not shown). The diminishing effect these requirements have on SNP identification likely explains the modest proportion of SNPs that intersect between both platforms.

Among those genome-specific SNPs found in both the 454/Sanger and Illumina data sets, only 677 (~1.3%) were in disagreement about the nucleotides representing the A- and D- genomes, leaving 49,535 (~98.7%) in agreement (Additional file 2). This last class of SNPs represents a high confidence category, though the SNPs identified only in the 454/Sanger or Illumina data sets have considerable internal support and also are likely reliable. For the cross-platform verified SNPs, we confirmed the presence of all A- and D-genome diploid SNP variants within the allotetraploid Illumina reads. This additional constraint of no further nucleotide evolution subsequent to polyploidization in either the diploid or allotetraploid lineages ensures that these SNPs almost certainly occurred prior to the formation of the allotetraploids. These SNPs can be used to diagnose both diploid *homologs* and allotetraploid *homoeologs*.

### Detection of nonreciprocal homoeologous recombination (NRHR)

**Figure 3:**
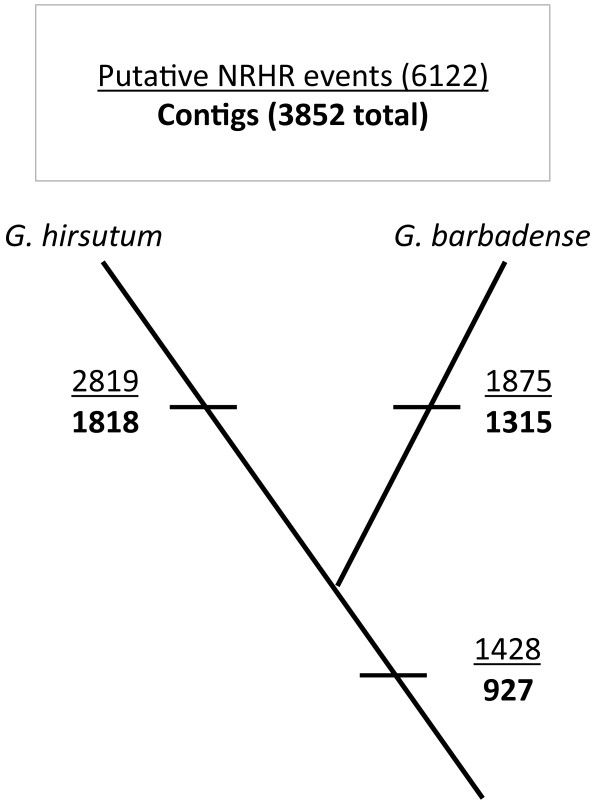
**Nonreciprocal homoeologous recombination (NRHR) events mapped onto a simplified *Gossypium *allotetraploid phylogeny.** Some contigs putatively contain multiple events. The total number of events is indicated in the upper value, while the aggregate number of affected contigs is indicated as the lower value (in bold). Values at the base of the phylogeny are those events shared by both species, which likely occurred in a common ancestor (although parallel mutation remains a possibility).

Genes doubled by allopolyploidy may, in principle, evolve independently or they may interact, thereby evolving in a “concerted” fashion, mediated by gene conversion or other forms of duplicate gene sequence homogenization. Salmon *et al.*[18], studied this phenomenon in allotetraploid cotton using an earlier release of the cotton transcriptome assembly. Following the methods outlined in Salmon *et al.*[18], we report an updated account of NRHR. This updated account includes a richer EST assembly, and also differs in that the current assembly includes sequences from a second allotetraploid species, *G. barbadense*. Because the two allotetraploid species used in this study (*G. hirsutum* and *G. barbadense*) share a common ancestor [4,5], detected NRHR can be mapped onto their phylogeny. Through this process we partitioned exchanges that occurred in only one allotetraploid lineage from those that occurred in their common ancestor (Figure 3).

A total of 6,122 unique NRHR were detected in the new assembly. Of these, approximately 46 % and 31 % are found only in *G. hirsutum* and in *G. barbadense*, respectively, whereas about 23% of the exchanges were found in both species (Figure 3). Interestingly, the unique set of 6,122 NRHR belong to only 3,852 contigs. Contigs with multiple exchanges may have experienced independent NRHR events, or alternatively, they may represent only one complex event, with several internal exchanges, that we have been unable to bridge together and resolve. This finding was initially reported by Salmon *et al.*[18], and its reiteration here with a much larger sample size provides evidence that certain duplicated genes or regions of the allotetraploid genome may harbor lengthy complex NRHR or could be “hotspots” of repeated exchange.

### Molecular evolution in diploid and allotetraploid cotton

The perspective provided by previous taxonomic and phylogenetic work [4,5] combined with the EST assembly generated here offers an unparalleled opportunity to explore the molecular evolutionary properties of genes in diploids and their allotetraploid derivatives. To assess the potential impact of polyploidy we compared sequence evolution between A- and D-genome orthologs in diploid cottons to that of A- and D-genome homoeologs in both species of allotetraploid cotton (as in Senchina *et al*. [13]). This was achieved by first isolating the A- and D-genome homoeologous reads from both allotetraploids, *G. barbadense* and *G. hirsutum*, (“A_T_” and “D_T_” hereafter) using EST sequences from the A- and D-genome diploid species as a guide (denoted by “A_2_” and “D_5_” hereafter). For each inter- (A_2_ vs. D_5_, A_T_ vs. D_T_) or intra-genomic (A_2_ vs. A_T_, D_5_ vs. D_T_) comparison we were able to study thousands of contigs and millions of nucleotides (Table 2 and Additional file 3), providing exquisite detail on the amount of divergence that has accumulated since the two diploids last shared a common ancestor, and since allotetraploid formation.

**Table 2:** Molecular evolution in diploid and allopolyploid cotton

**Comparision**	**Number of Contigs**^1^	**Total sites**	**Mean dS (Standard Dev.)**	**Mean dN (Standard Dev.)**	**Mean dN/dS (Standard Dev.)**
A_2_ vs. D_5_	12,517	13,867,911	0.036 (0.032)	0.009 (0.009)	0.308 (0.412)
A_T_ vs D_T_ - *G. barbadense*	7,151	9,843,949	0.040 (0.030)	0.009 (0.009)	0.289 (0.354)
A_T_ vs D_T_ - *G. hirsutum*	7,972	11,726,538	0.039 (0.031)	0.010 (0.010)	0.317 (0.411)
A_2_ vs A_T_ - *G. barbadense*	9,406	11,387,549	0.005 (0.01)	0.002 (0.003)	0.214 (0.313)
A_2_ vs A_T_ - *G. hirsutum*	9,997	12,421,297	0.006 (0.01)	0.002 (0.004)	0.263 (0.409)
D_5_ vs D_T_ - *G. barbadense*	9,236	12,237,260	0.009 (0.019)	0.003 (0.004)	0.240 (0.318)
D_5_ vs D_T_ - *G. hirsutum*	9,979	13,980,550	0.010 (0.014)	0.003 (0.005)	0.290 (0.409)

^1^See *Molecular evolution* section in the *Materials and Methods* for further detail about contig selection.

We isolated the coding regions (as predicted by ESTScan) from which we calculated the rates of synonymous (dS) and nonsynonymous (dN) divergence within and between species (Table 2). These two metrics can also be combined in as a ratio (dN/dS), which gives an indication of the selection pressures operating on the protein composition of a gene. Elevated dN/dS ratios (> 1) may be indicative of positive selection, though with low overall levels of divergence, as is seen in cotton, this interpretation must be viewed with caution as stochastic processes operating on a small number of total mutations can lead to spurious inferences of selection. Nonetheless, gene and genome duplication are thought to provide the opportunity for rapid gene evolution of one duplicate, so long as the other duplicate maintains sufficient ancestral functionality [29,30]. In the present context, we might expect to find an elevated dN/dS ratio between cotton A_T_ and D_T_ homoeologs, when compared to the dN/dS ratio of their A_2_ and D_5_ diploid progenitors*.*

**Figure 4:**
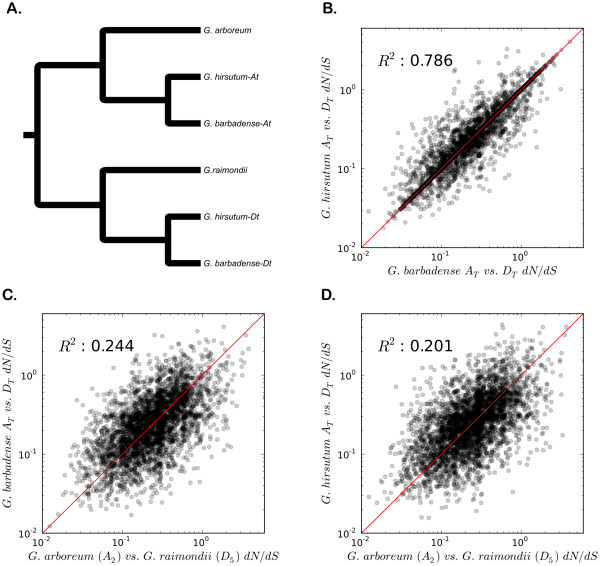
**A)*Homolog *and *homoeolog *relationships among *Gossypium *A- and D-genome diploids and allotetraploids.B**, **C**, and **D**) Pairwise plots of dN/dS ratios for between the A- and D-genomes of the allotetraploids *G. barbadense* and *G. hirsutum*, and the A- and D-genome diploids *G. arboreum* and *G. raimondii*. Axis are plotted on a natural log scale, with R^2^ values listed in the upper-left corner. Each dot represents one contig and the red line indicates the slope of a theoretical 1:1 relationship.

As shown in Table 2, we estimated dN and dS for anywhere from 7,151 to 12,517 contigs in pairwise comparisons among genomes. These values fall short of the total available contig counts listed in Figure 1 because some contigs lack a predicted protein-coding region or because the species in question fail to overlap one another within the contig alignment. Several conclusions are evident from these data, mirroring but also vastly extending similar results published by Senchina *et al*. [13] for a sample of only 48 genes: First, because the dataset is based on thousands of alignments in each comparison, it provides an accurate depiction of the ancestry of polyploid cotton. In this regard, the A_2_ diploid genome has a mean synonymous distance (dS) of 0.005 or 0.006 from the two A_T_-genomes whereas the comparable figure is 0.009 or 0.010 for D_5_ versus the two D_T_ genomes. From this we infer that modern *G. arboreum* is a better model (by about 50 %) of the actual genome donor of allotetraploid cotton than is *G. raimondii*, consistent with previous suggestions based on diverse data sources [3-5,13]. Second, though there is little protein evolution, that which exists is approximately twice as high (mean dN = 0.009) in inter-genomic contrasts (A_2_ vs. D_5_ or A_T_ vs. D_T_) genomes as it is for intra-genomic contrasts (A_2_ vs. A_T_ (mean dN = 0.002) or D_5_ vs. D_T_ (mean dN = 0.003)), as expected from the longer time period since A- and D-genome divergence (5 – 10 mya) than that between diploids and their corresponding genomes in the allotetraploid (1–2 mya) [4]. We also note that the approximately 50% increase in dN for D_5_ vs. D_T_ as compared to A_2_ vs. A_T_ mirrors the results for synonymous substitutions. Finally, we compared the mean dN/dS ratio for A_2_ vs. D_5_ relative to the two allotetraploid species (Figure 4). The mean dN/dS ratio for A_2_ vs. D_5_ is 0.308, while the two tetraploid species have A_T_ vs. D_T_ dN/dS ratios of 0.289 and 0.317, for *G. barbadense* and *G. hirsutum*, respectively (Table 2). In addition, Wilcoxon signed-rank tests on the dN/dS ratios show that *G. hirsutum* is significantly greater than either *G. barbadense* or A_2_ vs. D_5_ (both *P-values* < 0.001) while *G. barbadense* or A_2_ vs. D_5_ are statistically equivalent (*P-value* = 0.152). These data demonstrate that in the case of cotton, there is evidence for positive selection at the protein level following polyploidy in *G. hirsutum*, but not in *G. barbadense.* Of course, our data do not exclude the possibility that positive selection has occurred for *individual* genes based on functional advantages provided by amino acid substitutions in specific proteins in *G. barbadense*. We note that to the extent that such selection has existed, its footprints are most likely to be evident in the genes exhibiting high dN/dS ratios. To facilitate future exploration, we provide dN, dS, and dN/dS ratios at both the diploid and polyploid levels in Additional file 3. We also note the caveat that there is limited divergence experienced by cotton homoeologs, which results in stochasticity among dN/dS ratios derived from these small values. Given the many factors that influence the estimation of these ratios, as well as small sample size per taxon and the relatively minor difference in dN/dS ratios, it may be premature to conclude that the *G. hirsutum* truly has experienced accelerated protein evolution.

The overall low dN/dS ratios described above suggest considerable evolutionary constraint operating on both diploid and polyploid cotton genes. However, when comparing rates of sequence evolution between diploid and polyploid A- and D-genomes much of the evolutionary history is shared between parental species (A_2_ and D_5_) and their corresponding polyploid homoeologs (A_T_ and D_T_; see Figure 4a). To assess molecular evolution in the absence of this dependency, we phylogenetically polarized all nucleotide substitutions and filtered out only those that are specific to one lineage (Table 3). Though there are relatively few overall lineage-specific substitutions, we observe an increased level of DNA substitutions among both homoeologous genomes of both allotetraploid species relative to their diploid progenitors (all Exact Binomial test *P-values* < 0.001). The same overall pattern was found by Senchina *et al*. [13], though their results were not statistically significant due to a much smaller gene sample size. In conclusion, polyploidy in cotton has resulted in an increased level of DNA substitution in both allotetraploid genomes (Table 3), but this has only translated into an increased proportion of nonsynonymous mutations in *G. hirsutum* (Table 2).

**Table 3:** **Phylogenetically polarized lineage-specific substitutions for a)*Gossypium barbadense*, and b)*Gossypium hirsutum*allotetraploid genomes relative to their A- and D-genome diploid counterparts**

**Lineage**	**Number of Contigs**	**Total Nucleotides**	**Lineage-Specific Substitutions**
a) *Gossypium barbadense* lineage-specific SNPs relative to A- and D-genome diploids			
A_2_	4,863	3,755,712	3,211
A_T_	4,863	3,755,712	5,556
D_5_	5,750	3,735,542	4,754
D_T_	5,750	3,735,542	8,653
b) *Gossypium hirsutum* lineage-specific SNPs relative to A- and D-genome diploids			
**Lineage**	**Number of Contigs**	**Total Nucleotides**	**Lineage-Specific Substitutions**
A_2_	5,051	3,839,389	3,977
A_T_	5,051	3,839,389	6,412
D_5_	6,041	4,079,248	6,138
D_T_	6,041	4,079,248	10,393

## Discussion

### The cotton EST assembly

In the introduction we noted that because of cotton’s economic significance, it is important to understand both its transcriptome and its evolutionary history. Here we illustrate the interconnectedness of these two aspects of *Gossypium*, showing how insight into the transcriptome of allotetraploid cotton requires a sound phylogenetic and molecular evolutionary framework. It has become clear from *Gossypium* and investigations of other allotetraploids that genome doubling often entails violations of the assumption of additivity, *i.e.*, the allotetraploid genomes do not represent a simple combination of their diploid parents in their gene expression or content. For example, microarray analyses in allotetraploid cotton have shown that various tissues exhibit a pattern of parental genome expression described as “genomic dominance” (over-expression of one parental genome relative to the other when compared to the ancestral expression state of both diploid parents) [14,17,19]. Additionally, our analyses and those of Salmon *et al.*[18] have catalogued a surprising amount of NRHR in cotton allotetraploids that exhibit exclusive bivalent pairing at meiosis with no obvious cytogenetic hints at homoeologous exchanges. In light of these findings, it is important to understand the gene content similarities and differences among diploid and allotetraploid cotton. Toward that end, we present here a combined diploid and allotetraploid cotton transcriptome assembly, and utilize this assembly to assess broad patterns of genic evolution prior to and following polyploidy.

The cotton transcriptome assembly presented here contains 56,373 contigs. This assembly offers only a slight increase in contig number over the previous iteration [20], which had 51,107 contigs, but increases nucleotide coverage by nearly 7-fold. This expanded coverage makes it much easier to identify nucleotide substitutions. We used this added power and further supplementation by Illumina reads to document approximately 250,000 genome-specific SNPs and demonstrate the utility of these SNPs in diagnosing genic evolution and NRHR and in cotton allotetraploids. We expect that this collection of genome-specific SNPs will have broad utility in a diverse array of future studies spanning the full breadth of investigations in cotton that are enriched by genomic insight, including myriad applied objectives where SNP information will facilitate breeding, for example, or in fundamental investigations of genome-specific expression.

### A surprising level of non-independent evolution among homoeologs

With respect to the concept that allopolyploidy is accompanied by novel genetic or genomic phenomena of possible evolutionary significance, we build upon the approach of Salmon *et al*. [18] to show the extent of non-independent evolution among genic homoeologs in *Gossypium*. For comparison, in an initial survey of NRHR, Salmon *et al.*[18], attempted to determine the age of these events using a phylogenetic approach involving the 5 natural *Gossypium* allotetraploid species. Among six NRHR events, they found that five occurred in only one species, with the sixth event being ancestral. Here we extend this approach to examine tens of thousands of genes for conserved NRHR from two allotetraploid species (Figure 3). Based on these data, we report the surprising result that about 7% of all contigs in the assembly show evidence of NRHR in at least one of the two polyploidy species. As discussed by Salmon *et al*. [18], bioinformatic inferences of NRHR are subject to several possible sources of false positives, including assembly artifacts, sequencing error, and autapomorphic homoplasy subsequent to polyploid formation. However, Salmon *et al*. [18] established a 70% rate of validation using independent laboratory methods (PCR, cloning and sequencing), thus applying this false-positive rate we infer a true rate of NRHR in cotton to be approximately 5% of all contigs.

This high frequency of homoeologous contact is an astonishing result given the absence of prior cytogenetic observations that would have suggested this possibility, and apparently complete bivalent formation at meiosis. Furthermore, about one-quarter of all detected events are shared by both allotetraploids, indicating that they likely occurred in a common ancestor and have been retained to the present. The remaining exchanges occurred more recently, since the divergence of the two allotetraploid lineages, indicating that homoeologous exchange need not be restricted to the initial stages of allopolyploidization, during which time bivalent formation may not yet have been evolutionarily stabilized, but that it arises persistently on an evolutionary time scale. We also uncover approximately 50% more NRHR events in *G. hirsutum* compared to *G. barbadense*. The detection of NRHR relies on diagnostic reads, and thus detection should increase with deeper read sampling. Interestingly, *G. hirsutum* is only represented by about 7% more reads than *G. barbadense* in our assembly. For this reason, the increase in identified NRHR in *G. hirsutum* likely does not stem from greater read sampling alone. Instead, our results suggest that the rates of NRHR in the *G. hirsutum* and *G. barbadense* lineages have diverged following speciation.

Equally interesting is the possibility that NRHR events are not randomly distributed, instead that some genes maybe “hotspots” for NRHR. It will be fascinating to explore the genomic distribution of these hotspots once genome sequences become available. This approach might lead to insights into the mechanistic underpinnings of this form of “illegitimate intergenomic contact” following allotetraploidy in cotton. In addition, experimental analyses are required to assess whether any of the detected NRHR events have had physiological consequences and hence may be adaptively relevant.

### Molecular evolution in diploids and allotetraploids

The data set generated here provides an exceptionally detailed view of genic evolution in diploid and allotetraploid cotton. One of the key biological insights of the present study emerges from the observation of evidence for an increased rate of nonsynonymous to synonymous substitution between the A- and D-genomes in *G. hirsutum* when compared to the diploid A- and D-genomes or those in *G. barbadense.* We reiterate, however, the caveats that there is a limited timeframe for substitutions to have accumulated, which would tend to make these ratios sensitive a small number of substitutions. On the other hand, we do find strong evidence for a generalized increase in the nucleotide substitution rate in both allotetraploid genomes relative to their diploid progenitors. This same data set also provides a rich quantitative depiction of the genomic composition and history of allotetraploids, revealing the evolutionary relationships among the A_2_, D_5_, A_T_ and D_T_ genomes.

To assess whether the data could be mined to detect genic targets of selection, we estimated the rates of nonsynonymous to synonymous (dN/dS) mutation between the diploid A_2_ and D_5_ genomes and the allotetraploid A_T_ and D_T_ genomes. Overall, the vast majority of these dN/dS values are less than one. Regarding outliers, we find 943 contigs have a value greater than one between the A- and D-genome diploids, 470 between the A_T_ and D_T_ genomes in *G. barbadense*, and 532 between the A_T_ and D_T_ genomes in *G. hirsutum*. These contigs have experienced a greater amount of amino acid substitution than silent substitution (per site), a hallmark of positive selection, but we note again (as above) the high likelihood of false positives for dN/dS ratios when dealing with small numbers of substitutions. Among the genes that might be explored for selection, the largest outliers in each lineage are a cupin domain containing protein, a conserved plant protein of unknown function, and a putative phytocyanin protein (for the A- and D-genomes of *G. barbadense*, *G. hirsutum*, and the diploid A_2_ and D_5_ genomes, respectively). None of these rapidly evolving genes have yet been linked to phenotypic traits in cotton, but this is not surprising as relatively few cotton genes have a demonstrated phenotypic effect.

## Conclusions

In this manuscript we produce a new transcriptome assembly from diploid and allotetraploid cotton species and demonstrate several biological insights that can be derived from this resource. The large number of EST sequences generated for this study offer the most comprehensive view of the cotton transcriptome to date, and possibly the largest examination of the transcriptome from a polyploid plant and its diploid progenitors. By sequencing extant A- and D-genome diploids along with A- and D-genome containing allotetraploids, we are able to identify a vast number of SNPs that differentiate the allotetraploid A- and D-genomes. These SNPs can be used to explore molecular evolution within and between these genomes in both the presence and absence of polyploidy. Moreover, though cotton is a major crop, its genome is unsequenced. Our characterization of the transcriptome sheds light on the functional properties of the cotton genome.

Our results reveal an increased rate of nucleotide substitution in both polyploid lineages, though this increase only impacts the nonsynonymous to synonymous substitution ratio in *G. hirsutum*. We also uncover a surprising level of nonreciprocal homoeologous recombination (NRHR) between the allotetraploid A- and D-genomes. From these two findings we can conclude that genome duplication has impacted the mutation rate, and through long-term co-residency between the cotton A- and D-genomes, resulted in frequent illegitimate contact, resulting in homoeologous sequence conversion through nonreciprocal exchange.

## Methods

### Plant material and EST library construction and sequencing

454-FLX and Titanium ESTs were derived from various *Gossypium* species and tissue types (Table 1). RNA was independently extracted from each tissue source using a modified hot-borate method (Wilkins and Smart, 1996) and checked for integrity on Bioanalyzer (Agilent Technologies, Santa Clara, CA). Equimolar amounts of RNA from each extraction were combined into a single sample for cDNA library construction. cDNA libraries were constructed using SMART method (Clontech, Mountain View, CA) and the resulting amplified, double-stranded libraries were normalized using a double-strand nuclease (Trimmer, Evrogen, Moscow, Russia). To prevent poly-A (or poly-T) homopolyers in the 454 reads, we employed two strategies. The first strategy was applied to the FLX reads where a TypeIIS endonuclease was used to cleave 18–20 bp of transcript from a modified 3' SMART adapter (K. Delehaunty, personal communication). The second strategy, used for the 454 Titanium reads, employed PCR-suppression oligos to target particular regions in the transcript (5', internal, or 3'; [31]). 5’, internal, and 3’ transcript segments were pooled for cDNA sequencing of the *G. raimondii* sample. Only 5’ and internal segments were pooled for Titanium sequencing of *G. hirsutum* (Tx2094) and *G. barbadense* (K101 and Pima S6).

Sequencing was performed using 454 sequencing (454 Life Sciences, Branford, CT) at the Brigham Young University DNA sequencing center (FLX and Titanium) and Washington University (FLX). The reads have been made publically available through NCBI's Sequence Read Archive (Study #SRP001603). All publicly available Sanger reads were downloaded from GenBank (Feb. 2009) and filtered for duplicates, short ESTs (< 30 bp), and low-quality, vector, and low-complexity sequences using Lucy v. 1.20 [32].

### Assembly

All of the ESTs were combined to create a single, omnibus assembly of the *Gossypium* transcriptome that we have named Cotton46a where ‘46a’ refers to the assembly iteration. This assembly can be accessed and explored at our project website [33]. Sanger and 454 ESTs were assembled using the CLCBio Genomics Workbench (v. 3.7.1; CLC bio, Aarhus, Denmark) with the following parameters set for all collections of input sequence (similarity = 0.95, minimum length fraction = 0.5, insertion cost = 3, deletion cost = 3, mismatch cost = 2). Quality values were used for all 454 reads and as well as the *G. raimondii* Sanger reads, though most other Sanger reads retrieved from GenBank lacked quality scores. ESTScan v. 3.0.3 [26] was used to predict the coding sequences of each contig based on the codon preference matrix of *A. thaliana*.

### Assembly validation

We screened the contigs from our Cotton46a EST against well-annotated cloned and sequenced coding regions from the cotton diploid and tetraploid A- and D-genomes. In total we compared 14, 21, 19, and 13 genes and from A_2_, D_5_, and *G. hirsutum* A_T_ and D_T_, respectively (Additional file 4). We found no evidence for homoeologous miscategorization between A_T_ and D_T_. Among all accessions we analyzed 22,828 nucleotides and found 81 total substitutions, resulting in an error rate of 0.35 %. This error rate is likely inflated by allelic polymorphisms, as the exact genotypes used in our study were not necessarily the same as those retrieved from GenBank. For this reason this error rate should be considered a conservative upper-bound on the true error rate.

### Illumina mRNA-Seq generation and alignment to 454/Sanger reference contigs

Cotton fiber RNAs were extracted from *G. arboreum* (accession *cv*. AKA8401), *G. barbadense* (accessions K101 and *cv.* Pima S6), *G. hirsutum* (accessions *cv.* Maxxa and TX2094), and *G. raimondii* (accession unnamed) using a modified hot-borate protocol described in Hovav *et al.*[34]. RNA extractions were prepared for sequencing using the Illumina mRNA-Seq Sample Prep. Kit. Illumina sequencing (Illumina, Inc., San Diego, CA) was performed by the Iowa State DNA Facility. The reads have been made publically available through NCBI's Sequence Read Archive (Study #SRP001603). Reads were aligned to the 56,373 454/Sanger reference contigs using the *bwtsw* algorithm implemented by the BWA read-mapping software [35], leaving all parameters set to default.

### SNP detection

SNPs were extracted from the 454/Sanger contig assembly and the Illumina mRNA-Seq alignments in parallel. In the 454/Sanger assembly SNPs each contig alignment of the assembly consisted of overlapping reads. Where possible, a consensus sequence of each diploid genome was created from its respective reads. Similar to the parameters of CLCBio, a majority-rule at each position was used to determine all consensus sequences. Individual allotetraploid reads were then compared to the diploid consensus sequences so that they could be categorized as either belonging to the A- or D-genome. Consensus A- and D-genome allotetraploid sequences were then created from these allotetraploid ESTs and SNPs were identified based on differences between these two consensus sequences. For all consensus sequences, the major allele had a frequency greater than 90 %. This method was implemented using bioperl [36] and custom perl scripts.

In parallel, SNPs were extracted from the Illumina mRNA-Seq alignments using the *pileup* program from the SAMtools package [37]. All Illumina SNP calls were required to have a *pileup* SNP-quality score ≥ 20. Diploid A- and D-genome (*G. arboreum* and *G. raimondii*) specific SNPs and allotetraploid specific gene losses can confound homoeolog detection within allotetraploid cotton (see Figure 1 in Salmon *et al.*[18] for additional description). To overcome this problem, we checked if both the A and D parental SNP alleles were present among the allotetraploid reads. In so doing, we created 2 categories, Illumina SNPs with confirmed or unconfirmed presence in the allotetraploids. The Illumina SNPs with a confirmed presence are likely the result of shared ancestral substitutions within the cotton A- and D-genome lineages, and therefore have the highest reliability. The unconfirmed class of Illumina SNPs may include many *bona fide* SNPs shared by both the parental diploids and the allotetraploids, but we lack the evidence necessary to prove that they are not a result of diploid lineage-specific mutations or allotetraploid gene loss. For this reason we report only the confirmed class of Illumina SNPs.

### Pfam and Gene Ontology annotation

The search for protein domains and families within the current cotton assembly was performed with HMMER3 [38] using the manually curated Pfam-A protein database [39]. To broadly assess functional characteristics of the cotton assembly we assigned the Gene Ontology (GO) terms to contigs by converting their Pfam terms to GO terms using the pfam2go database [40,41].

### Sequence similarity searches

The assembled contigs were compared to the refseq_protein database from NCBI [42] using BLASTX to characterize the functional diversity within the transcriptome of cotton. BLASTX comparisons of the cotton transcriptome and *Arabidopsis thaliana* and *Carica papaya* (retrieved from Phytozome [24]) were also performed to compare gene content among these species.

### Nonreciprocal homoeologous recombination detection

The computational methods used to identify putative NRHR events are described in Salmon *et al.*[18], although the present study makes use of approx. 1.5 million *G. barbadense* ESTs that were not available to Salmon *et al.*[18]. Custom scripts were used to traverse the EST alignment for each contig, searching for *G. barbadense* or *G. hirsutum* reads that contain both A- and D-genome specific SNPs. Once identified, these reads were resolved into sets that support unique NRHR events. This was done by binning together all reads that share or extend a continuous NRHR SNP haplotype. Then, by tracking the species found within these sets we were able to identify events that were specific to either allotetraploid, as well as those that were shared by both species and thus likely occurred in a common allotetraploid ancestor.

### Molecular evolution

Using the same method of consensus sequence construction that was used for the 454/Sanger SNP analysis, the coding sequences of A_2_ and D_5_ were extracted from their respective consensus sequences using the coding frame predicted by ESTScan [26]. In addition for both *G. barbadense* and *G. hirsutum*, we extracted the homoeologous A_T_ and D_T_ genome sequences. After excluding genes with a putative NRHR event, synonymous and nonsynonymous substitutions were calculated in a pairwise manner using coding sequence alignments for each pair of species and the method of Nei and Gojobori [43]. These alignments were constructed using the maximum number of bases for each pairwise contrast, rather than the subset common to all species. Paired alignments less than 198 bp were not used and codons with ambiguous bases were removed. These parameters and variable amounts of sequence overlap between the individual consensus sequences within contigs resulted in differential numbers of alignments for overall diversity estimates. For these reasons, the number of contigs used for Table 2 and Table 3 is smaller than the total number of available contigs reported in Figure 1. All dN an dS estimates were corrected for multiple substitutions using the Jukes-Cantor correction implemented in bioperl [36]. The lineage-specific SNPs compiled in Table 3 were extracted from the alignments described above by phylogenetically polarizing substitutions to find those that occur only in one lineage, as assessed by comparing to the remaining lineages.

## Abbreviations

A2, Gossypium arboreum; AT, Allotetraploid Gossypium A-genome; contig, Contiguous DNA fragment; D5, Gossypium raimondii; dN, Nonsynonymous mutation rate; dS, Synonymous mutation rate DT, allotetraploid Gossypium D-genome; EST, Expressed sequence tag; NRHR, Nonreciprocal homoeologous recombination; SNP, Single nucleotide polymorphism.

## Competing interests

The authors declare that they have no competing interests.

## Authors’ contributions

JAU and JFW conceived and designed the study. JAU conducted EST sequencing and created the EST assembly. LEF and JFW generated the Illumina mRNA-Seq data. LEF and JAU analyzed the data. All authors drafted the manuscript and approve the final manuscript.

## Supplementary Material

Additional file 1 PDF file containing a figure that illustrates the contig counts for the top 30 Pfam categories among the Cotton46a EST assembly. Click here for file

Additional file 2 **Tab-delimited text file of all detected *Gossypium *A- and D-genome SNPs.** This file has been compressed with the open-source compression software bzip2. Click here for file

Additional file 3 **Tab-delimited text file of all dN and dS values, including intra- and inter-genomic contrasts.** This file has been compressed with the open-source compression software bzip2. Click here for file

Additional file 4 **PDF file containing a validation of EST assembly data using cloned and sequenced * Gossypium *coding regions retrieved from GenBank.** Click here for file
