# Supplementary figures and images for: Duplicate gene evolution, homoeologous recombination, and transcriptome characterization in allopolyploid cotton
Source: BMC Genomics. 2012 Jul 6;13:302. doi: 10.1186/1471-2164-13-302 (PMC3427041; doi:10.1186/1471-2164-13-302)

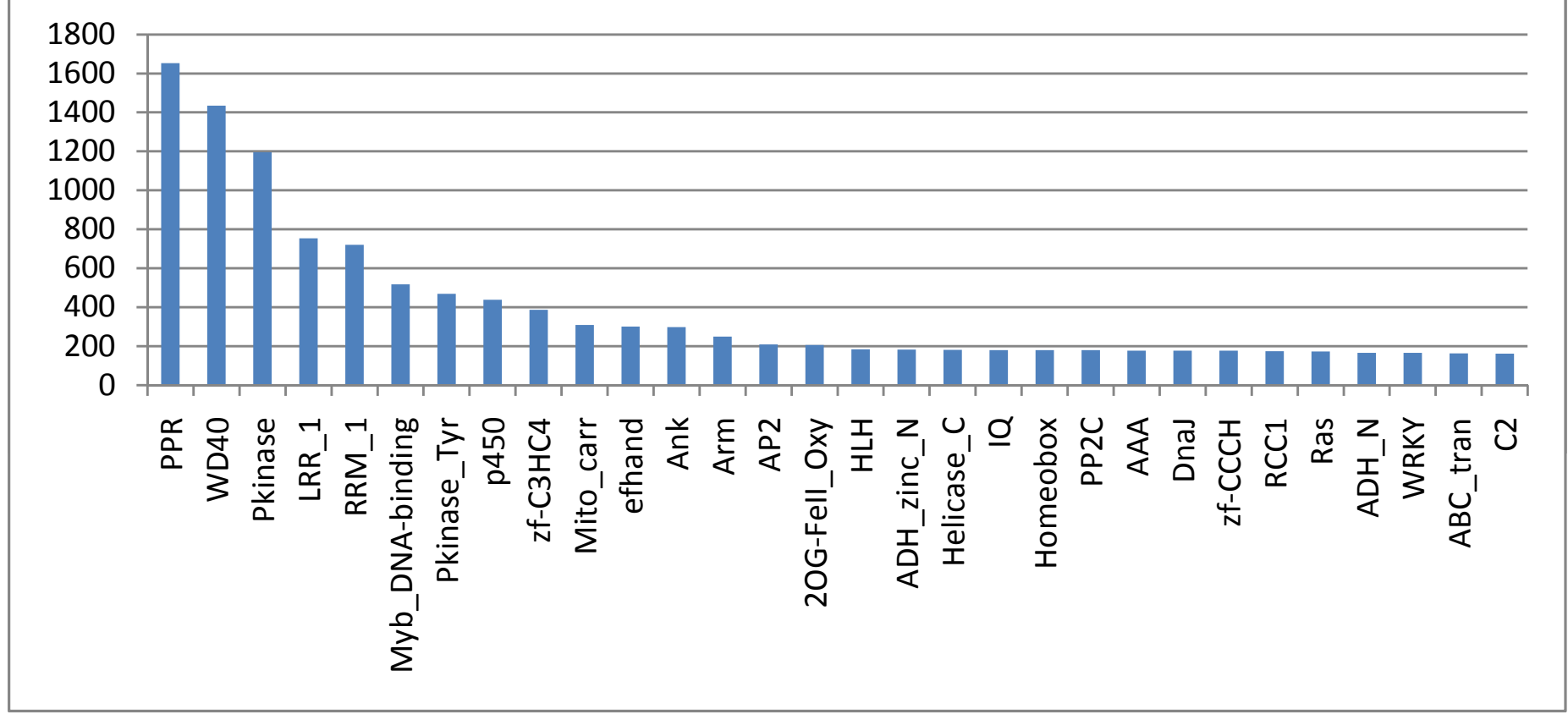

Supplement: Additional file 1 — PDF file containing a figure that illustrates the contig counts for the top 30 Pfam categories among the Cotton46a EST assembly. [file 1471-2164-13-302-S1.pdf]

Counts

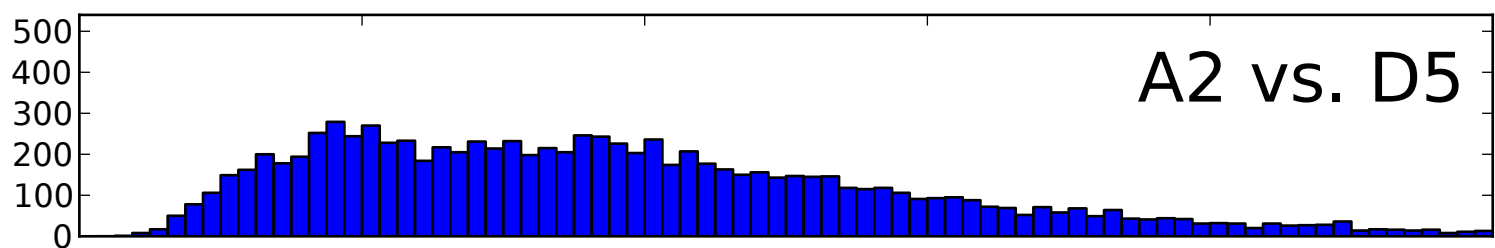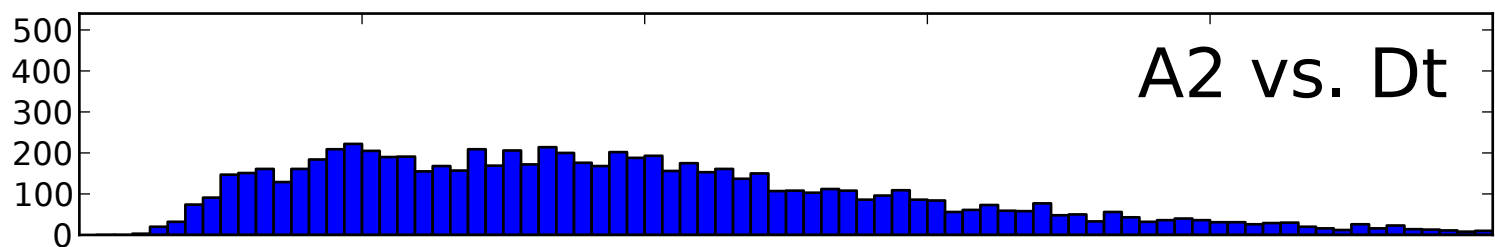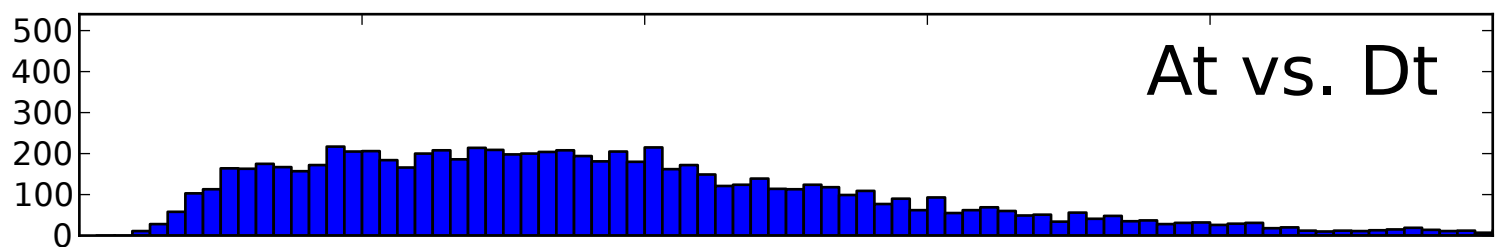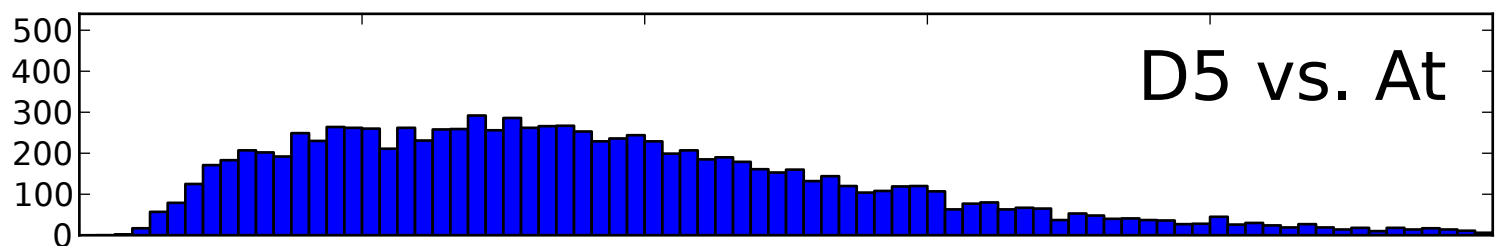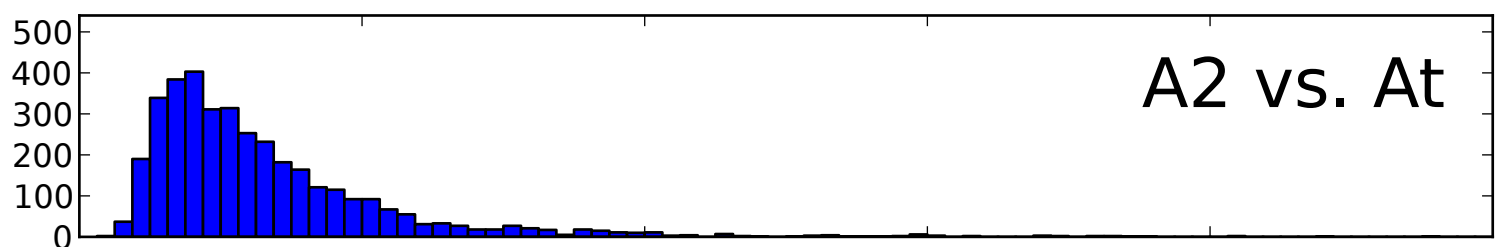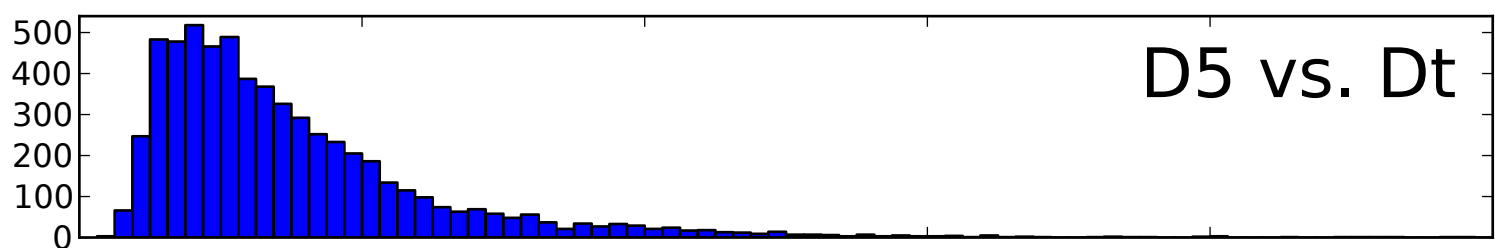

$dS$

Supplement: Additional file 3 — Tab-delimited text file of all dN and dS values, including intra- and inter-genomic contrasts. This file has been compressed with the open-source compression software bzip2. [file 1471-2164-13-302-S3.pdf]
