# Supplementary material for: Duplicate gene evolution, homoeologous recombination, and transcriptome characterization in allopolyploid cotton
Source: BMC Genomics. 2012 Jul 6;13:302. doi: 10.1186/1471-2164-13-302 (PMC3427041; doi:10.1186/1471-2164-13-302)
Supplement: Additional file 4 — PDF file containing a validation of EST assembly data using cloned and sequenced Gossypium coding regions retrieved from GenBank. [file 1471-2164-13-302-S4.pdf]

# A table summarizing the findings of the validation study conducted by comparing publically available sequences with the Cotton46a EST assembly

| gene name | genome | GenBank acc. | Cotton46a contig | blast evalue | mismatches | sites evaluated | %diff |
|---|---|---|---|---|---|---|---|
| B5 | A | AY116167 | Contig_21797_A | 0 | 2 | 489 | 0.00408998 |
| B5 | At | AY116169 | Contig_21797_At | 0 | 3 | 523 | 0.00573614 |
| B5 | D | AY116168 | Contig_54007_D | 0 | 1 | 523 | 0.00191205 |
| B7 | A | AY117055 | Contig_51818_A | 0 | 1 | 615 | 0.00162602 |
| B7 | Dt | AY117058 | Contig_51818_Dt | 0 | 0 | 615 | 0 |
| B7 | At | AY117057 | Contig_51818_At | 0 | 1 | 615 | 0.00162602 |
| B7 | D | AY117056 | Contig_51818_D | 0 | 0 | 615 | 0 |
| B8 | A | AY115496 | Contig_22616_A | 3.00E-62 | 0 | 120 | 0 |
| B8 | Dt | AY115499 | Contig_22616_Dt | 3.00E-53 | 1 | 109 | 0.00917431 |
| B8 | D | AY115497 | Contig_22616_D | 3.00E-62 | 0 | 120 | 0 |
| B8 | At | AY115498 | Contig_22616_At | 3.00E-62 | 0 | 120 | 0 |
| C3 | A | AY117061 | Contig_27205_A | 1.00E-157 | 1 | 284 | 0.00352113 |
| CesA1 | A | EU626444 | Contig_18567_A | 0 | 1 | 533 | 0.00187617 |
| CesA1 | Dt | AY632360 | Contig_18567_Dt | 0 | 1 | 533 | 0.00187617 |
| CesA1 | D | EU626442 | Contig_18567_D | 0 | 0 | 533 | 0 |
| CesA1 | At | AY632359 | Contig_18567_At | 0 | 1 | 533 | 0.00187617 |
| CesA2 | A | AY138240 | Contig_40547_A | 1.00E-123 | 2 | 256 | 0.0078125 |
| CesA2 | At | AY138242 | Contig_40547_At | 0 | 7 | 518 | 0.01351351 |
| CesA2 | D | AY138241 | Contig_40547_D | 0 | 0 | 518 | 0 |
| E11 | A | AF517656 | Contig_8458_A | 6.00E-65 | 5 | 144 | 0.03472222 |
| E11 | Dt | AF517659 | Contig_49048_Dt | 4.00E-60 | 1 | 120 | 0.00833333 |
| E11 | D | AF517657 | Contig_49048_D | 7.00E-77 | 0 | 144 | 0 |
| F12 | A | AY116157 | Contig_4987_A | 1.00E-137 | 0 | 246 | 0 |
| F12 | Dt | AY116160 | Contig_4987_Dt | 8.00E-136 | 1 | 246 | 0.00406504 |
| F12 | D | AY116158 | Contig_4987_D | 1.00E-137 | 0 | 246 | 0 |
| F12 | At | AY116159 | Contig_4987_At | 2.00E-121 | 1 | 223 | 0.0044843 |
| F4 | A | AY117100 | Contig_34253_A | 5.00E-113 | 0 | 205 | 0 |
| F4 | Dt | AY117103 | Contig_34253_Dt | 1.00E-110 | 1 | 205 | 0.00487805 |
| F4 | D | AY117101 | Contig_34253_D | 1.00E-110 | 1 | 205 | 0.00487805 |
| F4 | At | AY117102 | Contig_34253_At | 5.00E-113 | 0 | 205 | 0 |
| Myb1 | A | AY115504 | Contig_48656_A | 0 | 2 | 585 | 0.0034188 |
| Myb1 | D | AY115503 | Contig_31301_D | 0 | 2 | 335 | 0.00597015 |
| A1550 | A | AF201890 | Contig_25379_A | 6.00E-134 | 0 | 241 | 0 |
| A1550 | At | AF201891 | Contig_25379_At | 1.00E-131 | 1 | 241 | 0.00414938 |
| A1550 | D | AF201892 | Contig_37026_D | 2.00E-133 | 0 | 240 | 0 |
| A1623 | A | AF139474 | Contig_41536_A | 1.00E-59 | 1 | 120 | 0.00833333 |
| A1623 | Dt | AF139477 | Contig_41536_Dt | 6.00E-62 | 0 | 120 | 0 |
| A1623 | At | AF139475 | Contig_41536_At | 4.00E-75 | 1 | 154 | 0.00649351 |
| A1623 | D | AF139476 | Contig_41536_D | 6.00E-62 | 0 | 120 | 0 |
| A1751 | A | AF139437 | Contig_49337_A | 2.00E-157 | 1 | 284 | 0.00352113 |
| A1751 | At | AF139438 | Contig_49337_At | 0 | 8 | 807 | 0.00991326 |
| A1751 | D | AF139439 | Contig_49337_D | 0 | 5 | 456 | 0.01096491 |
| AdhA | A | EF457752 | Contig_36154_A | 0 | 1 | 414 | 0.00241546 |
| AdhA | Dt | EF457754 | Contig_36154_Dt | 0 | 0 | 414 | 0 |
| AdhA | At | EF457753 | Contig_36154_At | 0 | 1 | 414 | 0.00241546 |
| AdhA | D | EF457751 | Contig_36154_D | 0 | 6 | 414 | 0.01449275 |
| CelA1 | D | AF139442 | Contig_23048_D | 2.00E-105 | 0 | 193 | 0 |
| G1134 | Dt | AF139430 | Contig_4412_Dt | 5.00E-129 | 0 | 232 | 0 |
| G1134 | At | AF139428 | Contig_4412_At | 8.00E-125 | 2 | 232 | 0.00862069 |
| G1134 | D | AF139429 | Contig_4412_D | 5.00E-129 | 0 | 232 | 0 |
| CesABAC_02 | At | AY632359 | Contig_32982_At | 0 | 2 | 657 | 0.00304414 |
| CesABAC_02 | D | EU626442 | Contig_32982_D | 6.00E-70 | 0 | 134 | 0 |
| CesABAC_03 | At | AY632359 | Contig_48840_At | 0 | 3 | 612 | 0.00490196 |
| CesABAC_03 | D | EU626442 | Contig_48840_D | 2.00E-117 | 0 | 213 | 0 |
| CesABAC_07 | D | EU626442 | Contig_51983_D | 9.00E-120 | 0 | 218 | 0 |
| CesABAC_10 | Dt | AY632360 | Contig_43453_Dt | 0 | 0 | 519 | 0 |
| CesABAC_10 | At | AY632359 | Contig_43453_At | 0 | 1 | 424 | 0.00235849 |
| CesABAC_11 | Dt | AY632360 | Contig_4532_Dt | 0 | 6 | 739 | 0.00811908 |
| CesABAC_11 | At | AY632359 | Contig_4532_At | 0 | 5 | 489 | 0.01022495 |
| integralmembrane | At | EF457753 | Contig_6436_At | 6.00E-115 | 1 | 212 | 0.00471698 |
| oxidoreductase | D | EF457751 | Contig_2545_D | 0 | 0 | 448 | 0 |
| proteindisulfide | Dt | EF457754 | Contig_39884_Dt | 5.00E-164 | 0 | 292 | 0 |
| proteindisulfide | At | EF457753 | Contig_39884_At | 5.00E-164 | 0 | 292 | 0 |
| proteindisulfide | D | EF457751 | Contig_39884_D | 5.00E-164 | 0 | 292 | 0 |
| PS2 | Dt | EF457754 | Contig_39637_Dt | 2.00E-139 | 0 | 250 | 0 |
| PS2 | D | EF457751 | Contig_39637_D | 2.00E-139 | 0 | 250 | 0 |
| PS2 | At | EF457753 | Contig_39637_At | 2.00E-96 | 0 | 178 | 0 |
